# Sigma Routing Metric for RPL Protocol

**DOI:** 10.3390/s18041277

**Published:** 2018-04-21

**Authors:** Paul Sanmartin, Aldo Rojas, Luis Fernandez, Karen Avila, Daladier Jabba, Sebastian Valle

**Affiliations:** 1 Departamento de Ingeniería de Sistemas, Universidad Simón Bolívar, Barranquilla 080001, Colombia; arojas35@unisimon.edu.co (A.R.); lfernandez13@unisimon.edu.co (L.F.); 2 Departamento de Ingeniería de Sistemas, Universidad del Norte, Barranquilla 080001, Colombia; karena@uninorte.edu.co (K.A.); djabba@uninorte.edu.co (D.J.); sebastianvalle@uninorte.edu.co (S.V.)

**Keywords:** LLN, RPL, objective function, routing metric

## Abstract

This paper presents the adaptation of a specific metric for the RPL protocol in the objective function MRHOF. Among the functions standardized by IETF, we find OF0, which is based on the minimum hop count, as well as MRHOF, which is based on the Expected Transmission Count (ETX). However, when the network becomes denser or the number of nodes increases, both OF0 and MRHOF introduce long hops, which can generate a bottleneck that restricts the network. The adaptation is proposed to optimize both OFs through a new routing metric. To solve the above problem, the metrics of the minimum number of hops and the ETX are combined by designing a new routing metric called SIGMA-ETX, in which the best route is calculated using the standard deviation of ETX values between each node, as opposed to working with the ETX average along the route. This method ensures a better routing performance in dense sensor networks. The simulations are done through the Cooja simulator, based on the Contiki operating system. The simulations showed that the proposed optimization outperforms at a high margin in both OF0 and MRHOF, in terms of network latency, packet delivery ratio, lifetime, and power consumption.

## 1. Introduction

With the development of technology and the dissemination of smart technology, more and more devices are now connected to the Internet. This has unleashed the advancement of the Internet of Things (IoT) and the Low Power and Lossy Networks (LLN). The IoT is a concept in which every object or thing is connected to the Internet, allowing us to access them at any time and place, as long as an internet connection exists [1,2,3]. The nature of IoT is based on the use of wireless sensor networks. IoT was applied to the concept of intelligent cities, allowing cities to use sensor networks to build technological solutions that are efficient for the number of challenges that they face, such as the solution to problems of vehicular mobility [3,4].

Recently there has been a great interest in research and experimentation of wireless sensor networks, specifically in IPv6 routing for low-power consumption with high packet loss rates. As indicated in [5] over the years, the wireless network community has researched hundreds of new routing protocols aimed at different scenarios. However, most of the protocols’ requirements consist of energy and Quality of Service (QoS), for which they have been using aggressive standards or techniques for the autonomy of the batteries in the terminals (devices), the link quality, the network lifetime and the QoS offered to the user [1,2,3,6]. In today’s society, there is a great need to quickly access the diverse information offered by the Internet, emphasizing LLN with link state quality. However, in this field, there are still many topics of study that are still in constant evolution today [6,7,8,9,10,11].

Routing into such networks has been considered a challenge, which is why it is the focus of many research and global standardization groups. These groups lead directly to the design of the routing protocol IPv6 for LLN, called the “IPv6 Routing Protocol for Low-Power and Lossy Networks” (RPL) [12,13], standardized by the IETF and presenting itself as the front runner in the LLN.

The contributions of this work are the following:

- During the problem study of the link estimation, some implementation problems of RPL in the Contiki system were found. These implementation problems reduced the performance unnecessarily, which forced our experimental study to focus also in solving these problems.
- SIGMA-ETX was designed and implemented to allow RPL to test the link quality through bidirectional traffic with only simple modifications, without violating the standard, and to verify its improvement in performance through test simulation.

The rest of this paper is organized as follows: Section 2 defines the RPL protocol, Section 3 presents related works, Section 4 presents the proposed SIGMA-ETX metric model, Section 5 explains the design and simulation of the network, Section 6 describes the result of the simulation, Section 7 discusses RPL issues, and Section 8 offers concluding remarks.

## 2. RPL Protocol

The IPv6 Routing Protocol for Low Power and Lossy Network (RPL) is based on a distance vector and was standardized by IETF under ROLL (Routing Over Low Power and Lossy Networks) review. There are two types of nodes in an RPL network: the sink (or receiver) node gathers information throughout the network, and the origin or issuer node collects data from sensors to send them to the sink node. 

RPL uses the Destination-Oriented Directed Acyclic Graph (DODAG) concept [6,14,15,16] to define its topology. For this reason, the following types of control messages should be defined:

- DIO: DODAG Information Object
- DIS: DOGAG Information Solicitation
- DAO: Destination Advertisement Object
- DAO-ACK: Destination Advertisement Object Acknowledgement

The DODAG is constructed downwards from the sink node, using DIO messages that contain information such as the root node identifier and the objective function, which is used when selecting parents for each node [17].

The nodes in the network keep transmitting DIO messages until all nodes have received and sent their ranks. Once this is done, every node selects their preferred parent, which is the node through which they send their packets. It is possible for each node to store information about other nodes in the network, in order to change a preferred parent if needed. The new DIO messages are sent periodically through the Trickle Timer algorithm to update the network.

The DAO message is sent by all nodes to the root node and closes the route between the sender and the root node. The DAO-ACK message can be requested by a node to guarantee the delivery of the DAO. Lastly, the DIS message is used by new nodes that join the network to request a DIO and thus keep the topology up-to-date.

The rank of a node in RPL is represented as a scalar number which depicts the location of that node within the DODAG. Each node in the network has a rank value: the rank of a node defines the relative single position from a node with respect to a sink node of the DODAG, and it is calculated based on the distances relating to its neighbors. The rank can be calculated as a function of link metrics and may consider other properties, such as limitations [6,11]. In this process of DODAG network forming, each node selects multiple nodes as possible parents and the objective function defines how RPL nodes select their parent to improve routes within a RPL Instance.

An objective function defines how an RPL node selects the optimized route within an RPL instance, based on metrics and routing restrictions. It provides specific criteria of optimization, such as minimizing hop count, ETX route, latency, etc. RPL forms a DODAG based on the objective function. The OF guides RPL in the selection of parents and candidate parents. It is also used by RPL to calculate the rank of a node. All upstream traffic is transmitted through the preferred parent.

Objective Function zero (OF0) selects the route to the root using a minimal number of hops. This can be achieved by comparing the parents’ ranks. Cooja uses a rank of 16 bits in units of 256 (min_hoprankinc) that allows a maximum of 255 hops. The ETX metric of a wireless link is the expected number of transmissions needed to send a packet through the link. The ETX Objective Function uses the ETX metrics while calculating the shortest path.

On the other hand, the Objective Function (OF) in RPL is used by the nodes, in order to select forwarding nodes on an application-defined routing metric. RPL has two OFs. Among the functions standardized by IETF we find the OF0, which is based on the minimum count of hops, and the MRHOF, which is based on the expected transmission count (ETX). However, when the network becomes denser or the number of nodes increases, both OF0 and MRHOF introduce long hops, which can which can develop into a bottleneck that restricts the network. The adaptation is proposed to optimize both OFs through a new routing metric.

In this paper, the performance of RPL in various traffic scenarios was investigated through experimental measurements [14,15]. In addition, an adaptation of a specific metric for the RPL protocol in the objective function MRHOF is presented.

## 3. Related Works

Traditionally, only one metric is used to select the route. However, this metric cannot satisfy the Quality of Service (QoS) requirements [18,19] of different applications. Therefore, some proposals [12,16,20] present methods that combine metric types to satisfy the needs of the applications. For example, the Hop Count (HC) [9,10] selects the route with the lowest number of hops, while the Expected Transmission Count (*ETX*) selects the route with the least number of expected transmissions [21,22,23]. 

The *ETX* metric is the number of transmissions a node expects to make to a destination in order to successfully deliver a packet, and it can be represented by the following formula:

$$ETX=\frac{1}{Df\times Dr}$$

where *Df* is the measured probability that a packet is received by the neighbor, and *Dr* is the measured probability that the acknowledgment packet is successfully received [10].

The high number and sheer diversity of applications in networks such as WSN imposes challenges and different requirements with respect to the criteria of delay, loss, and energy. In terms of energy, Patrick et al. [24] define Residual Energy (RE) in the RPL protocol, where RE metric represents the available energy at one node. In LLNs, nodes are usually battery-powered, therefore, it is necessary to choose the nodes with the most available energy for routing. In another proposal published by Zhao et al. [15,16], the authors describe a novel energy-efficient region-based routing protocol called ER-RPL that uses the ETX metric for calculating the rank. Additionally, Thulasiraman [25] presents an RPL protocol for the mesh-based multi-gateway Advanced Metering Infrastructure (AMI) of the Smart Grid, where they define the Expected Transmission Time (ETT): this metric is based on ETX according to the packet size and the link transmission capacity. This technique enables the distribution of a small packet followed by a big packet to each neighbor, after which each neighbor measures the arrival time between the two packets and informs the sender of that time. Finally, the sender calculates the link capacity by dividing the size of the biggest packet delay by the smallest packet delay [26]. 

In other research [27] an “optimization model to minimize the expected end-to-end transmission time in wireless mesh networks”, this model is based on the Weighted Cumulative ETT (WCETT) metric, has been proposed by da Silva et al. WCETT focuses on fixed nodes of local networks, where the goal is to select a high-throughput path to avoid any loss of information between the source and its destination. The metric assigns weights to the individual links, which are called Expected Transmission Time (ETT). WCETT is a value that increases depending on the number of links in the network where ETT is an estimation of the end-to-end delay that a packet experiences while traveling along a path between two nodes [28,29]. In Table 1, the target performance of these metrics are introduced.

In [30], the authors have presented the design of a path weight structure for wireless routing and some guidelines that identify the specific properties that a routing metric must have. This work describes the Bamer metric, which is a proposed structure to capture power consumption for more reliable communications of wireless links with packet loss. Bamer is suitable to source routing protocols combined with the discovery of routes based on floods (e.g., DSR Protocol) or the Dijkstra algorithm. If using Bamer with routing protocols based on the Bellman-Ford algorithm (e.g., DSDV), the routing protocol is not optimal, but it is still coherent and free of loops. Therefore, Bamer is still usable, although its performance can be degraded [24].

Fotouhi et al. propose the mRPL protocol, a solution to cope with mobility as one of the challenging issues for future IoT applications [31]. This research integrates a proactive hand-off mechanism (dubbed smart-HOP) within RPL [31]. An energy-based RPL objective function is proposed [32], which is based on the remaining energy of each node. Other research in [33] describes the problems of RPL when implemented for mostly downward traffic, referring to it as an upward-oriented protocol. A modified protocol called DT-RPL is proposed for diverse traffic requirements, including both upward and downward traffic. The protocol exploits downward packets to update the ETX metric values in the child nodes transmitted in the RPL messages. Other research for Downward routing in RPL is proposed by Simon Duquennoy et al. [34], where they classify the different causes of packet loss and present a set of reliability mechanisms that eliminate or mitigate all causes of packet losses.

Khallef et al. in [35] introduces a new OF based on a Non-Linear Length (NL-OF), which takes into account any number of metrics and constraints for QoS routing. NL-OF ensures that each path in the DODAG respects the input constraints. The NL-OF can be used to meet the requirements of sensible applications, such as real-time applications. Other research in [36] proposes a Queue Utilization-based RPL (QU-RPL) that improves the load balancing and congestion traffic in LLN networks. QU-RPL is designed for each node to select its parent node considering the queue utilization of its neighbor nodes as well as their hop distances to an LLN border router (LBR).

Another protocol is described in [37] where the authors evaluate the performance of the P2P-RPL variant and compare it with normal RPL. P2P-RPL differs from RPL, in that the traffic flows in pre-calculated DAGs in the latter, whereas in P2P-RPL, the best-quality route is calculated for any source-destination node pair. The authors create a simulated P2P-RPL environment in NS3 and find that the Packet Delivery Ratio in P2P-RPL is higher than on RPL, due to the specific routes selected for the particular source-destination pairs, instead of always going through the root of the DODAG as in normal RPL.

In [20], Wei Xiao et al. propose a new metric for the RPL protocol called Per Hop ETX (PH-ETX). PH-ETX calculates the best routing path, taking the average of the ETX values between each node along the path to avoid the problem of a long, single hop. To improve the quality of service in LLN networks, one of the most widely used techniques is the ETX metric, which focuses on connection states at the network layer level. Among measurements previously mentioned, some of them are specific for a best parent selection in each node. However, PH-ETX defers the responsibility of best parent choosing to ETX, and then uses it to select the best path for routing.

## 4. Proposed SIGMA-ETX Metric

The ETX metric tries to choose the best wireless link for data transmission. However, because the sum of ETX values between each node along a route are used to choose the best route, the number of hops of a path are more important than the quality of transmission at the time of making the decision. In this way, the chosen route with a lower ETX summation tends to be the one with the fewer number of hops. The problem is more evident when the network is dense (the number of nodes increases); there exists the possibility for a better routing path to have more nodes, thus making its ETX value greater than that of a single, long hop. Those long hops will develop into bottlenecks for the whole network.

The routing metric called SIGMA-ETX was created to solve the problem mentioned above. SIGMA-ETX is a combination of the minimum hop count metric and ETX, where the best route for the routing is calculated with the standard deviation of the ETX values between each node.

**Figure 1:**
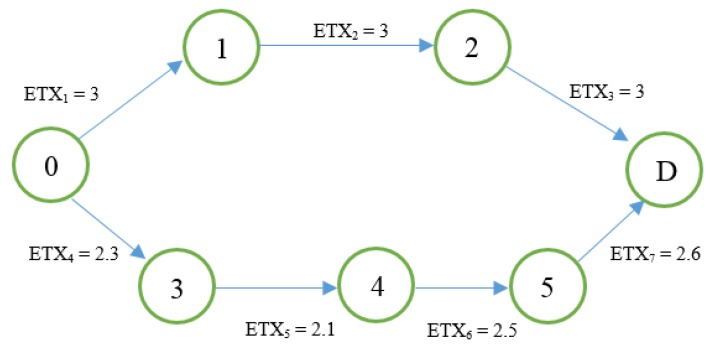
Sample PH ETX.

In Figure 1, from source node 0 to destination node D, there are two accessible paths. The calculation of the ETX values for Route 1 (0–1–2–D) is as follows:

*ETX*_0*D*1_ = *ETX*_1_*+ ETX*_2_ + *ETX*_3_*=* 3 + 3 + 3 = 9

For the route 2 (0–3–4–5–D).

*ETX*_0*D*2_ = *ETX*_4_ + *ETX*_5_ + *ETX*_6_ + *ETX*_7_ = 2.3 + 2.1 + 2.5 + 2.6 = 9.5,

*ETX*0*D*2 > *ETX*0*D*1

According to the traditional *ETX* metric, route 1 is the best path between node 0 and D, but it is probable that a bottleneck will occur, because the *ETX* value is too large between node 0 and node 1 (or node D and node 1). Therefore, in most cases, this solution would not be the best routing path. PH-ETX compares [17] the routes, taking into account the average of *ETX*. For the presented network model, this would be the result:

(1) $$PH-ETX_{0D1}=\frac{ETX_{1}+ETX_{2}+ETX_{3}}{3}=3$$

(2) $$PH-ETX_{0D2}=\frac{ETX_{4}+ETX_{5}+ETX_{6}+ETX_{7}}{4}=2.37$$

Suppose there are two candidate paths in the network. Path 2 has *n* nodes, and all of them follow the Gaussian distribution. Path 1 has *m* nodes, where one of them has a long hop:

(3) $$\sum_{i=1}^{n}ETX_{i}>\;\sum_{j=1}^{m-1}ETX_{j}+ETX_{0},$$

(4) $$n\bar{ETX}>\left(m-1\right)\bar{ETX}+ETX_{0}$$

(5) $$\frac{\sum_{j=1}^{m-1}ETX_{j}+ETX_{0}}{m}>\;\frac{\sum_{i=1}^{n}ETX_{i}}{n}$$

The objective function will choose Route 2, based on the statistical principle. Therefore, the new metric could avoid the long hop problem. However, in deployed dense networks, the average calculation does not satisfy all possible situations in order to avoid long hops; consequently, some packets are lost. This is the case in situations where the average value of the *ETX* metric is close between possible paths. When this happens, the PH-ETX objective function applies the shortest path to choose the appropriate route, which brings us back to paths with longer hops. The following example shows a tie situation between *ETX* averages:

To solve this problem, the minimum hop count metric and *ETX* are combined, creating a new routing metric called SIGMA-ETX, where the best route is calculated with the standard deviation of the *ETX* values between each node.

**Figure 2:**
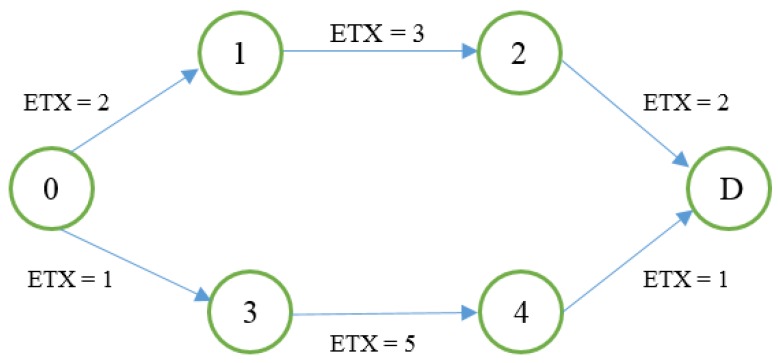
Example of tie between *ETX* averages.

To calculate SIGMA-ETX for the scenario in Figure 2, the standard deviation of each route is needed:

(6) ∑(R1−ETX)=2+3+2=7

(7) ∑(R2−ETX)=1+5+1=7

The summation of both routes is a tie, so the objective function tries to use the number of hops but they are also the same. However, route 2 has a very long hop that can become a bottleneck. Since their *ETX* summations and number of hops the same, the average strategy does not distinguish the long hop due to both averages being equal:

(8) $$Avg\;\left(r1\right)=\frac{7}{3}=2.3$$

However, with the standard deviation:

(9) $$\sigma =\sqrt{\frac{1}{n-1}\overset{n}{\underset{i=1}{\sum }}{\left(ETX_{i}-\bar{ETX}\right)}^{2}}$$

Applied for route 1,

(10) $$\left(R_{1}\right)=\sqrt{\frac{1}{3-1}\overset{3}{\underset{i=1}{\sum }}{\left(ETX_{i}-2.3\right)}^{2}}$$

$$\left(R_{1}\right)=\sqrt{\frac{1}{2}}\;\left[{\left(2-2,3\right)}^{2}+{\left(3-2,3\right)}^{2}+{\left(2-2.3\right)}^{2}\right]$$

$$\sigma \left(R_{1}\right)=\sqrt{\frac{1}{2}}\;\left[\left(0.09\right)+\left(0.49\right)+\left(0.09\right)\right]$$

(11) $$\sigma \left(R_{1}\right)=0.57$$

And applied for route 2

(12) $$\sigma \left(R_{2}\right)=\sqrt{\frac{1}{3-1}}\overset{3}{\underset{i=1}{\sum }}{\left(ETX_{i}-2.3\right)}^{2}$$

$$\sigma \left(R_{2}\right)=\sqrt{\frac{1}{2}\left[{\left(1-2.3\right)}^{2}+{\left(5-2.3\right)}^{2}+{\left(1-2.3\right)}^{2}\right]}$$

$$\sigma \left(R_{2}\right)=\sqrt{\frac{1}{2}}\;\left[{\left(-1.3\right)}^{2}+{\left(2.7\right)}^{2}+{\left(-1.3\right)}^{2}\right]$$

(13) $$\sigma \left(R_{2}\right)=2.30$$

where *n* is the number of nodes (i.e., hops) and $\bar{ETX}$ the average per route, then:

$\sigma \left(R_{1}\right)=0.57$ standard deviation of route 1

$\sigma \left(R_{2}\right)=2.30$ standard deviation of route 2.

Route 2 has a higher standard deviation. This is a more complete way of recognizing that route 1 is more stable, having no longer hop.

### 4.1. SIGMA-ETX CDF

**Figure 3:**
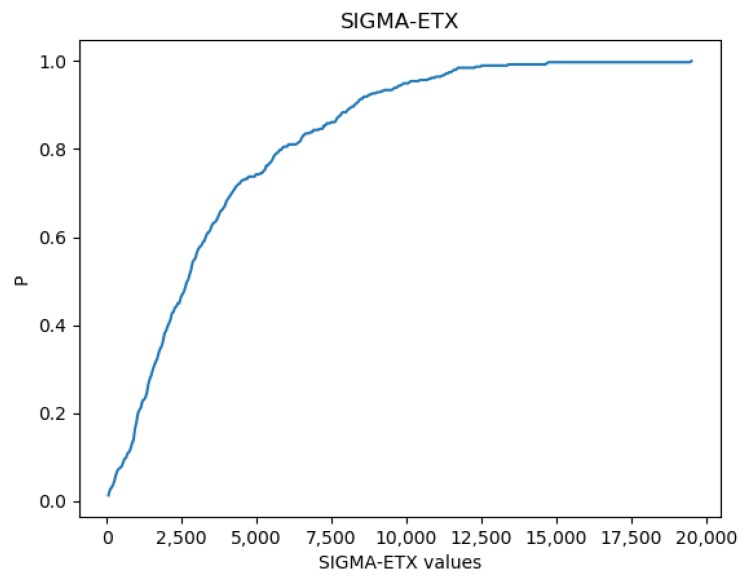
SIGMA-ETX CDF.

Figure 3 presents the Cumulative Distribution Function (CDF) of SIGMA-ETX for routes with two hop counts based on the 40 nodes simulation. The figure shows that 80% of all two-hop routes in the network have a SIGMA-ETX value of 6000 at most.

Following the definition of SIGMA-ETX, as the sigma value approaches zero, the homogeneity of the ETX values in the path increases, and thus the probability of long hops decreases. Figure 3 can be interpreted as follows: 40% of the results of the metric have a value of up to 2500, while 100% of the results can reach values of up to 20,000. This confirms the high disposition of the network to create long hops that are more unstable.

### 4.2. General Scheme

**Figure 4:**
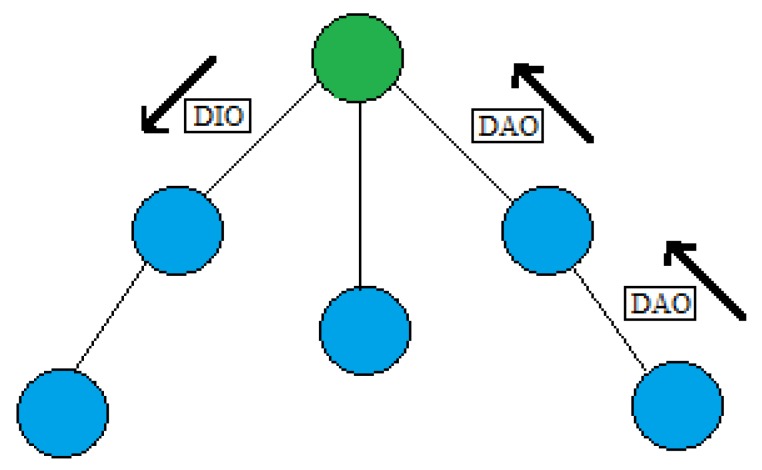
DAO Format.

In an RPL environment, the DODAG root sends a DIO message that is propagated and received by every node in the network. Every node in the DODAG sends a DAO message (to the root) with relevant information to create the network topology (See Figure 4).

To calculate SIGMA-ETX, some fields are added to the DAO messages containing the ETX values of the sender node and its neighbors. This information is transported from the sending node to the DODAG root.

**Figure 5:**
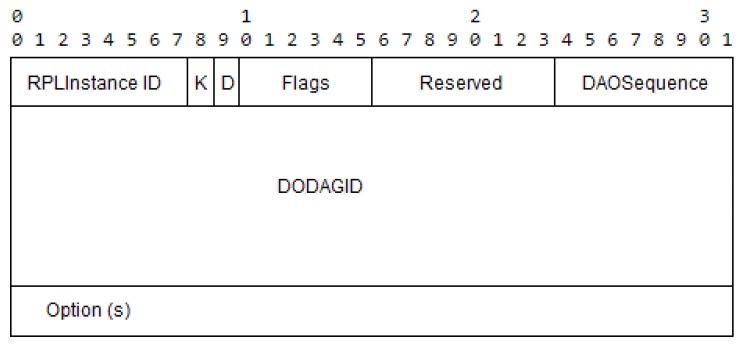
DAO format.

With the topology information obtained from the DAO messages, control messages can be sent from the root of the RPL instance, by explicitly specifying in each message what will be the hops that each message will take. Two optional blocks were added to the option fields (See Figure 5) of the RPL DAO messages: 

RPL_OPTION_PATH_LENGTH: 

Contains a single 32-bit field indicating the length of the path from the DAO source to the root of the RPL instance.

RPL_OPTION_PATH_MEMBER:

Contains the prefix of the IPv6 address of a single node in the path from the DAO source to the root of the RPL instance, and the ETX value of that individual hop.

The root uses a new option in the DAO-ACK messages to relay the calculated SIGMA-ETX values to the nodes. The DAO-ACK message has a similar structure as the DAO message, allowing extra options to be appended as needed.

**Figure 6:**
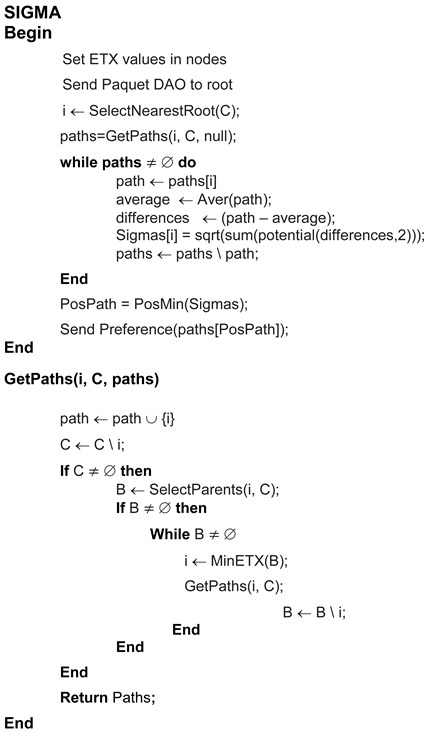
Pseudocode scheme.

As Figure 6 shows, the nodes calculate their ETX values with respect to their neighbors, and then send the DAO messages to the DODAG root. Once this information reaches the root, it is used to calculate the SIGMA values for each path, so that the preferred routes can be identified.

## 5. Design and Simulation of the Network

The simulation of the network wasdone with a single sink node, using a topology in order to distribute the nodes in a square area with a side L = 500 m. Also, the RPL network was designed using OF0, MRHOF, PH-ETX, and the SIGMA-ETX metric, proposed for its comparison with simulations under different numbers of nodes, with a receiver node or additional sink. Following this, an RX value of 100% was used and the behavior of RPL was investigated with regard to packet delivery ratio and power consumption. The main RPL default parameters used in the simulations are shown in Table 2.

The simulation is based on the IPv6 architecture and uses IEEE 802.15.4 at the PHY and MAC layer to form LLNs. With the IEEE 802.15.4 standard, a device can be set to operate in the beacon-enabled mode or the non-beacon enabled mode. This work of this paper is based on the un-slotted non-beacon-enabled mode with CSMA/CA, in the 2.4 GHz ISM band, which is the default configuration of ContikiOS. On top of that, it uses 6LoWPAN, RPL, and IPv6 to provide end-to-end two-way communication to each node.

### 5.1. Parameterization of Scenarios

In this section, each experimental test that was performed with the Cooja simulator is briefly described in order to evaluate the multi-hop wireless sensor network. First, tests were performed to evaluate the losses caused in the transmission and later the latency experienced by the shipments of packets in the network. Following this, different scenarios were simulated to evaluate the efficiency of the multi-hop wireless sensor network; the results are discussed.

In order to comply with the above, the simulations were implemented in an experimental environment, with different number of nodes (20, 40, 50 and 100) as scenarios, which were deployed in a 500 m × 500 m zone, with a number of 200 packets delivered, and transmission range of 150 m.

**Figure 7:**
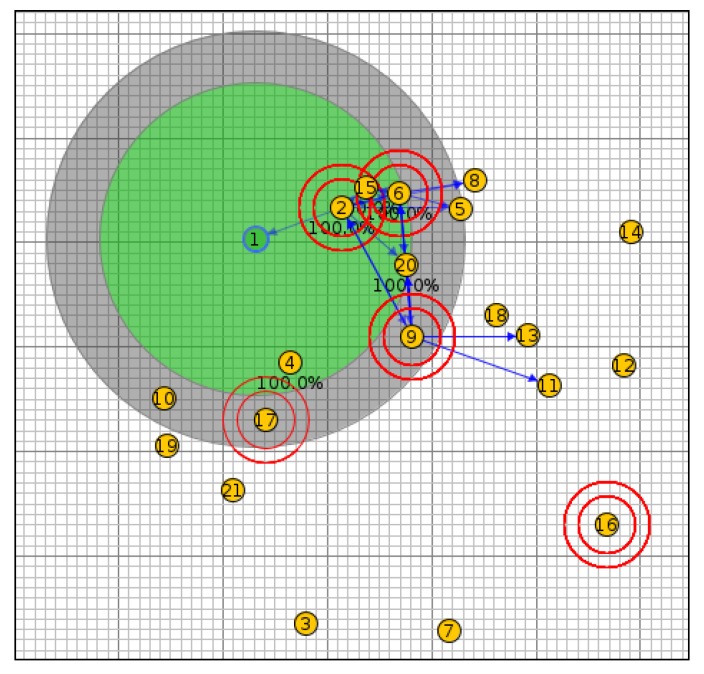
Scenario I with 20 nodes.

**Figure 8:**
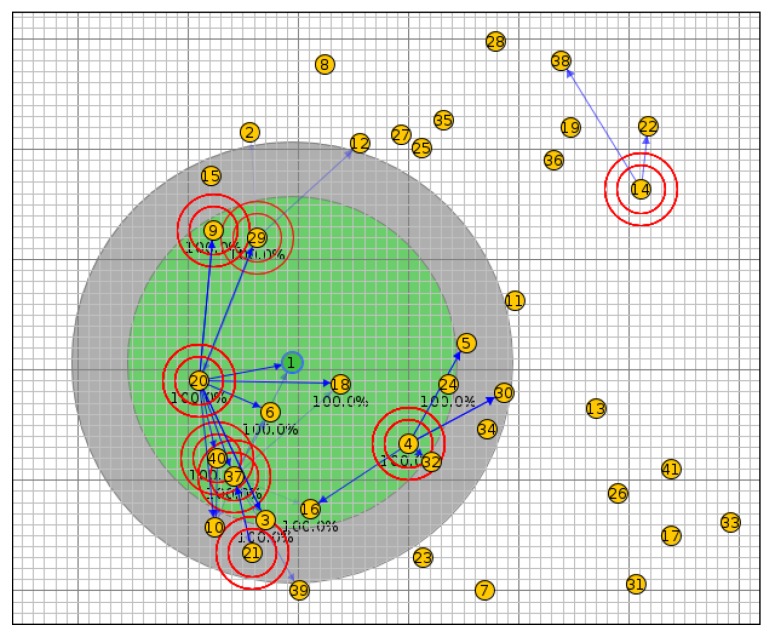
Scenario II with 40 nodes.

**Figure 9:**
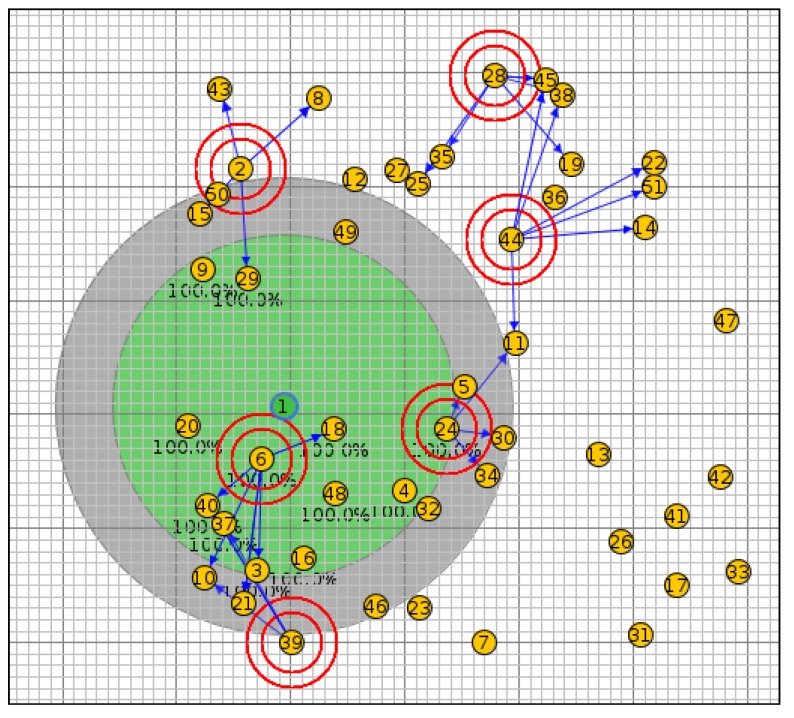
Scenario III with 50 nodes.

**Figure 10:**
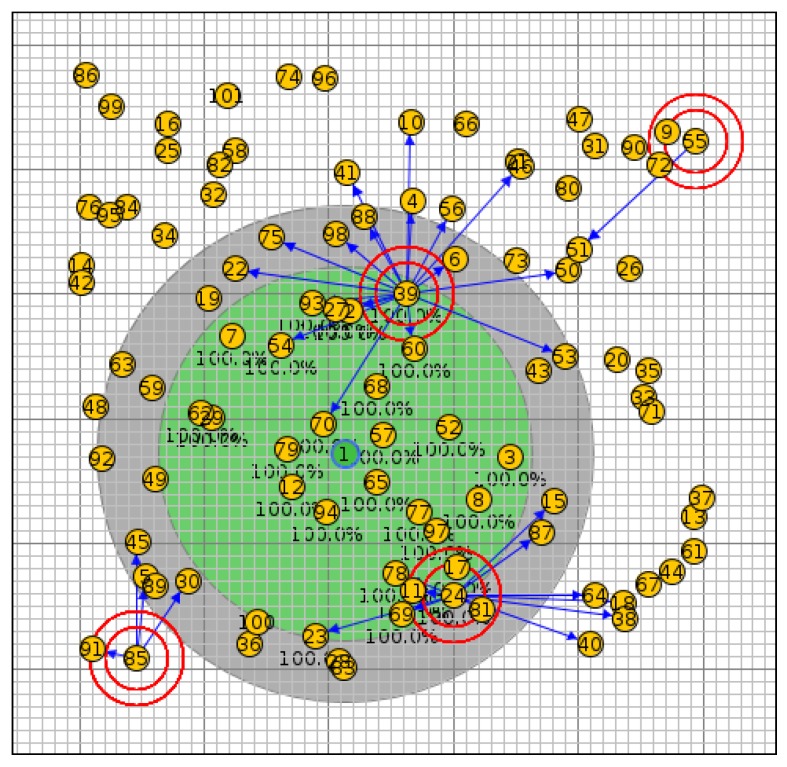
Scenario IV with 100 nodes.

In scenario I, the network was simulated with 20 nodes using the OF0, MRHOF, PH-ETX and SIGMA-ETX metrics, all of them with the same configuration (see Figure 7). Figure 8 shows scenario II with 40 nodes; scenario III with 50 nodes and scenario IV with 100 nodes are presented in Figure 9 and Figure 10.

## 6. Results

After validating the technical configurations in each scenario, the simulation performance were made. The purpose of this section was to analyze the results of the simulations. A special coding was made in the Cooja simulator to obtain the complete results of each scenario, which can be consulted in the information shown below.

By creating a simulation with Cooja in Instant Contiki 3.0, a comparison was made between the newly proposed objective function SIGMA-ETX and these three functions: OF0, MRHOF, and PH-ETX. Several simulations were performed, between 20, 40, 50 and 100 client nodes and 1 root or receiver node.

### 6.1. Latency of the Network

**Figure 11:**
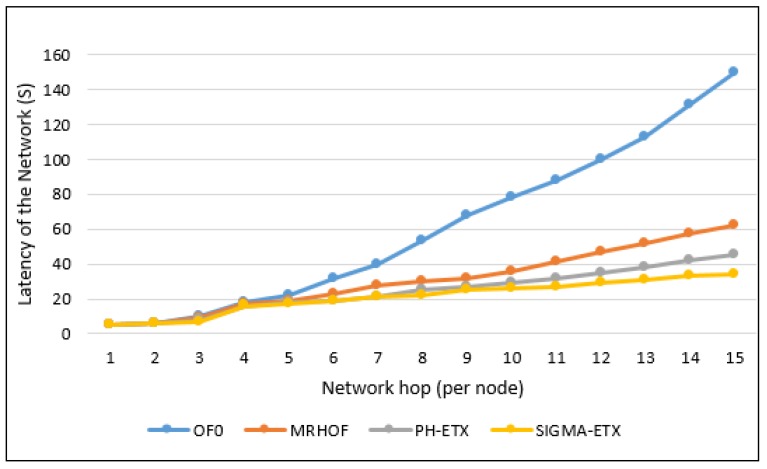
Latency of the network.

OF0 is presented in relation to ETX, MRHOF, PH-ETX, and SIGMA-ETX, the latter of which has a significantly higher latency percentage. According to Figure 11, it can be seen that SIGMA-ETX is superior in performance to OF0 and MRHOF. As for PH-ETX, the performance difference with the proposed metric is not as significant as with OF0 and MRHOF, but it is still noteworthy. To accomplish that, SIGMA-ETX makes an optimal route selection, preferring paths with fewer long hops, and thus making it superior to the other three metrics. In terms of SIGMA-ETX, detailing the results at the 15th hop, significant differences can be noted against OF0 (23.33%), MRHOF (56.45%) and PH-ETX (83.33%).

**Figure 12:**
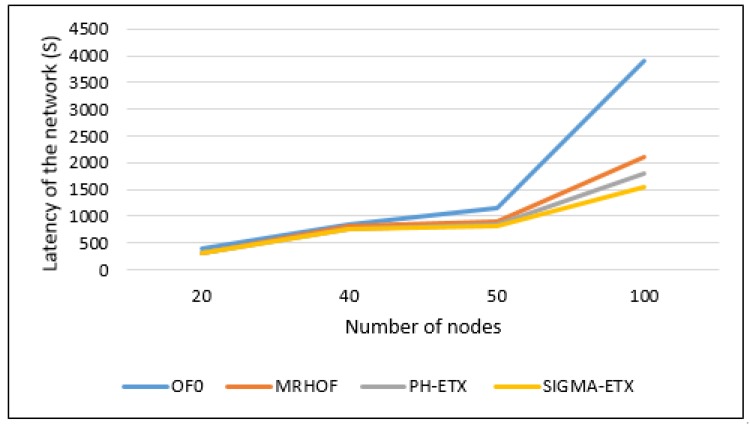
Latency with different nodes.

Based on the Figure 12, the network latency does not change much between the four methods for the 20 nodes scenario, due to the low number of nodes. The 40 nodes scenario does not show much difference, but it becomes more apparent in the 50 and 100 nodes scenarios. In terms of percentages, SIGMA-ETX comes first among the metrics, with a better latency performance, beating the next best metric by 3% in the 100 nodes scenario. While this might seem of little significance, it suggests a better quality projection in denser networks.

### 6.2. Packet Delivery Ratio

**Figure 13:**
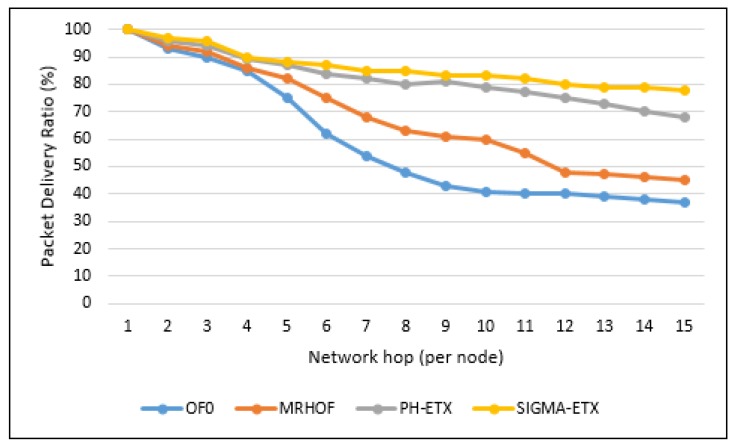
Packet delivery ratio.

The packet delivery average is calculated with the equation PDR = (Total Packets Received/Total Packets Sent) × 100. In Figure 13, the packet delivery ratio of SIGMA-ETX is slightly better than PH-ETX, and much greater than OF0 and MRHOF, demonstrating the validity of SIGMA-ETX. Putting 15th hop results in context, the difference against SIGMA-ETX regarding packet delivery is 47.44% with OF0, 57.69% with MRHOF, and 87.18%. with PH-ETX.

**Figure 14:**
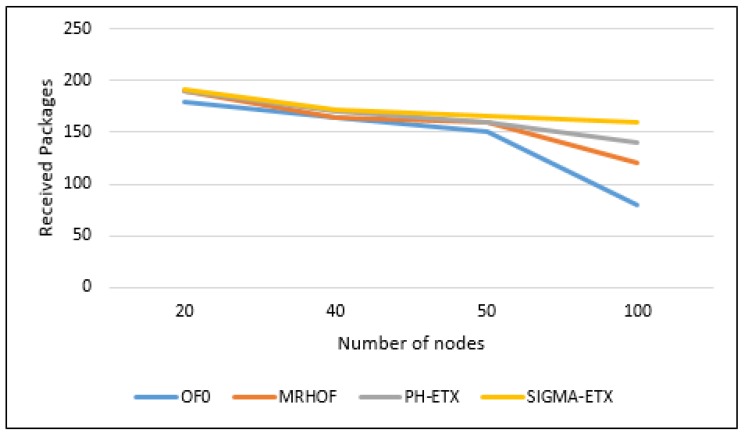
Packet delivery ratio.

Figure 14 shows that with 200 packets sent, ETX, MRHOF, PH-ETX and SIGMA-ETX have similar packet loss in both the 20 nodes and 40 nodes scenarios. However, for 50 and 100 nodes, we can see that PH-ETX has a lower packet loss, delivering 80% of the sent packets in the entire network.

As shown in Figure 14, while the tendency of all metrics is to increase the packet loss in denser scenarios, SIGMA has a 10% increase in packet delivery ratio with respect to PH-ETX, and a much higher margin compared to the other metrics.

### 6.3. Energy Consumption

In order to estimate the energy consumption with precision, the percentage of radius over time was used, since the radius depends on the current consumption in the sensor nodes.

**Figure 15:**
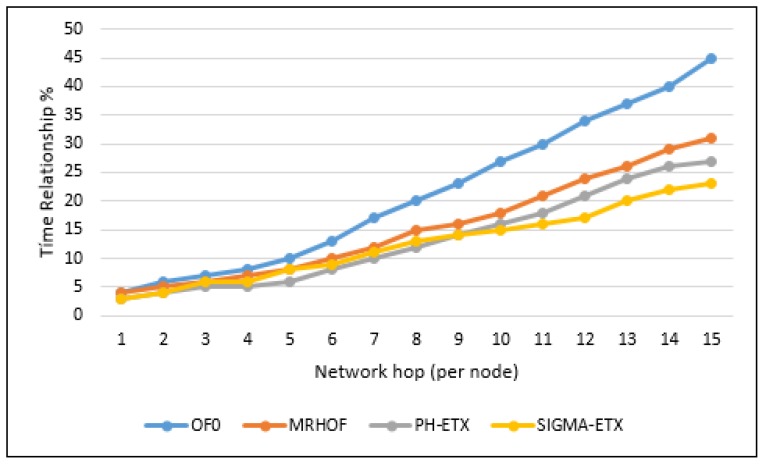
Energy consumption.

As shown in Figure 15, when the network is small, because of complex calculations, SIGMA-ETX can consume more energy, but when the network expands, SIGMA-ETX can maintain better connections than PH-ETX, making total consumption lower. Regarding energy consumption, results show OF0 reaches 51.11% of SIGMA-ETXs results, MRHOF reaches 74.19%, and PH-ETX reaches 85.19%.

### 6.4. Lifetime

The network lifetime is defined as the time during which the network is operational. It ends when a node runs out of energy and can no longer send packets.

**Figure 16:**
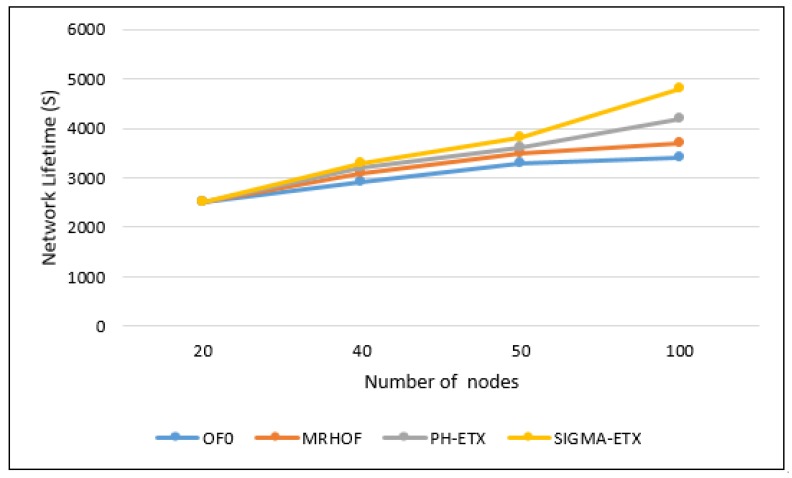
Network lifetime.

It is clearly shown in Figure 16 that with the optimization of SIGMA-ETX, the lifetime of the network is higher than with the other metrics. This is due to the selection process of SIGMA-ETX, which tries to identify the best route by preferring paths without long hops, thus avoiding extra packet loss and latency. The prevented packet losses result in less retransmissions and in turn, lower power consumption. Particularly, the results in a 100-node scenario shows evidence that OF0 only achieves 70.83% of SIGMA-ETXs lifetime performance, MRHOF only 77.08%, and PH-ETX 87.50%.

## 7. Discussion

LLN networks have a big growth and applicability potential in IoT; this explains why they have attracted the attention of many manufacturers to invest in different technologies that facilitate the interaction between different devices (nodes) at the data link level. These technologies take maximum advantage of IPv6 addresses (which are a plus for LLN), each node having its own IP address. This means LLNs will be exponentially scalable, and the RPL protocol will be a central piece in their development.

One of the challenges of RPL in LLN is unquestionably security. Many of the vulnerabilities of sensor networks will no doubt make the transition to IoT, particularly with RPL attacks like Sinkhole, HELLO Flood, Wormhole and Clone ID, which occur using intruder nodes. In any case, care must be taken, as many IoT devices (nodes) may already have operating systems that are vulnerable to other types of attacks, such as Androids.

On the other hand, many manufacturers (Cisco, Microchip, Atmel, among others) have implemented and tested RPL in their products, obtaining good results. They have made RPL available to the public, which is why it is much easier to perform tests in real IoT scenarios with the RPL protocol. Additionally, some of these manufacturers have taken the risk and used ContikiOS as the operating system for their products (devices), including thus the RPL protocol, considering there is also an implementation of RPL for other open source operating systems (e.g, Linux).

However, one must admit that the RPL implementation in the Contiki operating system has some room for improvement. The implementation has some issues and is not complete according to the description found in the RFC 6550, which is why we believe there are still many things to improve and test to bring RPL in the IoT to its full potential.

## 8. Conclusions

In this paper, the adaptation and implementation of a routing metric for the objective function in the RPL protocol was proposed. Once the appropriate state-of-the-art metric was defined, it was possible to observe the recent contributions in the subject by other researchers, which allowed the analysis of the operation of the RPL protocol to understand and modify the protocol, by adapting the link state metric in its objective function.

LLN scenarios were configured with parameters close to reality; this is how the SIGMA-ETX metric was implemented, guaranteeing its operation by comparing its implementation with the performance of the objective functions OF0, MRHOF, and PH-ETX with SIGMA-ETX. This demonstrated that SIGMA-ETX is more efficient with networks containing large number of nodes per unit of area in the LLN routing in terms of quality of service. In addition, the results of SIGMA-ETX were compared with the results of other research, in terms of latency, detailing the results at the 15th hop. Significant differences could be noted against OF0 (23.33%), MRHOF (56.45%) and PHETX (83.33%). 

The packet delivery ratio of SIGMA-ETX is slightly better than PH-ETX, and much greater than OF0 and MRHOF, demonstrating the validity of SIGMA-ETX. Putting 15th hop results in context, the difference with OF0 is 47.44%, with MRHOF reaching 57.69% and with PH-ETX reaching 87.18% against SIGMA-ETX regarding packet delivery.

Regarding energy consumption, results show OF0 reaches 51.11% of SIGMA-ETX’s results, MRHOF reaches 74.19%, and PH-ETX reaches 85.19%. The prevented packet losses resulted in less retransmissions and also lower power consumption. Particularly, the results in a 100-node scenario shows evidence that OF0 only achieves 70.83% of SIGMA-ETX’s lifetime performance, MRHOF only 77.08%, and PH-ETX 87.50%.

On the other hand, we suggest that SIGMA-ETX could be combined with other metrics to calculate a better route. This can be done because ContikiRPL was designed to easily allow this kind of implementation. Additionally, the ContikiRPL code can be found in SourceForge and GitHub.

In the future, it is necessary to research the relationship between other types of Quality of Service (QoS) metrics (e.g., link reliability and others), SIGMA-ETX, and the useful life of the node, because the complexity of the processes and the variability of these types of networks require the contrast of SIGMA-ETX, as opposed to metrics in specific applications of LLNs. Estimating the relation of these aspects will allow future researchers to define parameters to use this result. Additionally, we are working to apply load balance in SIGMA-ETX to improve the functionality of RPL and to apply a Software Defined Network (SDN) to RPL networks.

## Figures and Tables

**Table 1:** Metrics.

Metric	Target Performance
HC	Communication latency and energy consumption reduction (indirectly)
ETX	Reduction of required frame retransmissions
RE	Expansion of network lifetime
PFI	Reduction of network-layer losses
ETT	Assignment of weights to the individual links
WCETT	Focus on fixed nodes of local networks

**Table 2:** Summary of simulation parameters.

Parameters	Value
Area	500 m × 500 m
Boot delay per node	1 sg
Number of nodes	20, 40, 50 and 100
TX Range	150 m
Network Protocol	RPL
Standardized Objective Functions	OF0, MRHOF
Objective Functions proposed by other authors	PER HOP ETX
Standard for PHY and MAC layer	IEEE 802.15.4
Transmission Mode	Storing Mode
Topology	Random
Number of packets	200
Model of radius	UDG
ContikiOS Mote Type	Cooja Mote
